# The relationship between self-efficacy and prenatal depression in Chinese pregnant women: a parallel latent growth curve model

**DOI:** 10.1186/s12888-025-07623-4

**Published:** 2025-11-28

**Authors:** Xiaoxiao Mei, Ranran Mei, Funa Yang, Shuhan Li, Limei Kang, Youjin Lei, Zengjie Ye

**Affiliations:** 1 School of Nursing, Guangzhou Medical University, Guangzhou, China; 2 School of Nursing, The Hong Kong Polytechnic University, Hong Kong, China; 3 Breast Oncology Department, Guangzhou Institute of Cancer Research, the Affiliated Cancer Hospital, Guangzhou Medical University, Guangzhou, China; 4 Department of Obstetrics, Jiangmen Central Hospital, Jiangmen, China; 5 Department of Obstetrics, Guangzhou University of Chinese Medicine First Affiliated Hospital, Guangzhou, China

**Keywords:** Adverse childhood experiences, Prenatal depression, Parallel latent growth curve model, Pregnant women, Self-efficacy

## Abstract

**Background:**

Prenatal depression is a significant public health concern that affects both mothers and their infants. Self-efficacy has been identified as a protective factor against prenatal depression; however, the longitudinal dynamics between these constructs, particularly concerning adverse childhood experiences, remain underexplored.

**Methods:**

This study utilized a prospective cohort design involving 721 pregnant women from the Be Resilient to Postpartum Depression study in China. Assessments were conducted during the first (T1), second (T2), and third trimesters (T3) from January 2022 to August 2022. Data were collected using the Adverse Childhood Experience scale, the General Self-Efficacy Scale, and the Hospital Anxiety and Depression Scale. Data analysis was conducted using parallel latent growth curve models.

**Results:**

The findings indicated a significant correlation between baseline self-efficacy and baseline prenatal depression levels (*β*=-0.267, *P* < 0.01). Additionally, the change rate of self-efficacy was strongly associated with the change rate of prenatal depression (*β*=-0.434, *P* < 0.01). Notably, baseline prenatal depression significantly predicted the change rate of self-efficacy (*β* = 0.115, *P* = 0.034). In the multigroup parallel latent growth curve models, the change rate of prenatal depression levels was associated with the change rate of self-efficacy (*β*=-0.424, *P* < 0.01) and was predicted by baseline self-efficacy levels (*β*=-0.023, *P* = 0.031) in the group without adverse childhood experiences. Conversely, within the adverse childhood experiences group, baseline prenatal depression levels significantly predicted the change rate of self-efficacy (*β* = 0.227, *P* = 0.049).

**Conclusion:**

Enhancing self-efficacy may mitigate the impact of prenatal depression, particularly among women with adverse childhood experiences.

**Supplementary Information:**

The online version contains supplementary material available at 10.1186/s12888-025-07623-4.

## Introduction

Prenatal depression is a depressive episode that occurs without psychotic features and represents a substantial public health concern [1, 2]. It is characterized by symptoms such as sadness, diminished concentration, sleep and appetite disturbances, as well as ongoing irritability and anxiety [3]. A recent review analyzed 182 studies conducted across 50 countries, involving 197,014 pregnant women, and reported a global pooled prevalence of prenatal depression at 20.7% (95%CI 19.4–21.9%) [4]. In contrast, another review focused on 31 studies conducted in mainland China, encompassing a total of 83,173 participants, and found a higher pooled prevalence of 22% (95%CI 17–28%) [5]. This condition may lead to pregnancy complications, a heightened likelihood of postpartum depression, and negative developmental consequences for children [6–8]. Considering its prevalence and harmful effects, identifying the factors that contribute to prenatal depression is vital for developing effective strategies for pregnant women.

## Self-efficacy and prenatal depression

Self-efficacy is defined as the belief in one’s capability to execute behaviors necessary for achieving specific performance outcomes [9]. Extensive research has demonstrated that women with diminished self-efficacy in managing their pregnancy are more likely to experience symptoms of prenatal depression during both early pregnancy [10] and late pregnancy [11, 12]. Pregnant women with greater self-efficacy tend to show better emotional regulation, enhanced problem-solving skills, and increased engagement in health-promoting behaviors, all of which are associated with lower depressive symptoms during pregnancy [13, 14].

Conversely, prenatal depression can adversely affect pregnant women’s self-efficacy, leading to feelings of helplessness when confronted with challenges [15]. Besides, Research indicates that women experiencing higher levels of prenatal depression report lower self-efficacy regarding diabetes management during pregnancy, suggesting that depressive symptoms can undermine their confidence in managing health-related tasks [16]. This interaction creates a negative feedback loop that underscores the bidirectional link between self-efficacy and prenatal depression.

However, the dynamics of how self-efficacy influences the trajectory of prenatal depression over time remain poorly understood. Most existing studies have focused on the connection between self-efficacy and depression from the third trimester to the postpartum period [17, 18], leaving a significant gap in our understanding of how self-efficacy affects the trajectory of prenatal depression from the first trimester to the third trimester.

## The moderated role of adverse childhood experiences

Adverse childhood experiences (ACEs) refer to traumatic events encountered by individuals under the age of 18, including physical and emotional neglect, different types of abuse, exposure to domestic violence, mental health problems, family incarceration, separation, and substance abuse [19, 20]. Studies consistently indicate that ACEs are negatively related to self-efficacy [21, 22] and positively related to depression [23, 24]. For instance, one study specifically highlighted that emotional abuse and neglect, common forms of ACEs, diminish self-efficacy by undermining individuals’ confidence in coping with difficulties and achieving goals [22]. Moreover, empirical evidence reveals that psychological and physical abuse, along with neglect, are significant predictors of prenatal depression scores among pregnant women, with ACEs accounting for 49.1% of the variance in depressive symptoms [25]. Consequently, ACEs undermine self-efficacy, thereby diminishing their protective effects and exacerbating the link between low self-efficacy and elevated depression. This interaction could affect how self-efficacy and prenatal depression are related over time in pregnant women [26, 27]. Gaining insight into these dynamics is essential for designing targeted interventions that strengthen self-efficacy and promote better mental health among pregnant women affected by ACEs.

## The current study

In light of these considerations, the present study seeks to explore the longitudinal connection between self-efficacy and prenatal depression in pregnant women, with particular attention to whether ACEs serve as a moderator in this relationship. To achieve this, we will employ a parallel latent growth curve model (LGCM). This approach allows for the simultaneous modeling of the growth trajectories of two or more variables, facilitating an analysis of how their initial levels and rates of change are interrelated over time [28]. Specifically, we hypothesize that:

1. Baseline self-efficacy levels correlate with the baseline depression levels.
2. The changes in self-efficacy correspond to shifts in depression levels from the first to the third trimester.
3. Baseline self-efficacy levels predict the change in depression levels, while baseline levels of depression predict the change in self-efficacy.
4. ACEs will moderate the longitudinal linkages between self-efficacy and prenatal depression, resulting in distinct patterns for women with and without ACEs.

## Methods

### Design and participants

This prospective cohort study was conducted as part of the Be Resilient to Postpartum Depression (BRPD) program. Recruitment took place from January 2022 to January 2023, targeting pregnant women receiving prenatal care at two hospitals in Guangdong province. Three evaluations took place during the prenatal phase: the first trimester (before 13 weeks, T1), the second trimester (between 22 and 28 weeks, T2), and the third trimester (after 32 weeks, T3). The criteria for inclusion included: (1) being 18 years or older; (2) confirmed pregnancy through ultrasound; and (3) fluent Mandarin communication skills. The exclusion criteria included: (1) a confirmed mental illness diagnosis by medical personnel; and (2) termination of pregnancy during the mid-term.

### Sample size

The sample size for this study was determined using a structural equation model sample size calculator [29], as LGCM represents a specific application of the structural equation model for analyzing change over time [30]. The calculation factored in a small to medium effect size (0.2), with 6 constructs and 22 observed measures. Targeting a statistical power of 0.8 and a significance level of 0.05, the adjusted sample size, accounting for a 10% non-response rate, was approximately 443 pregnant women.

### Measurements

#### Demographic variables

Based on the existing studies [31, 32], the demographic characteristics were assessed via questionnaire at T1, including age, education level, work status, monthly average household income, and parity status (multiparous vs. nulliparous).

#### Adverse childhood experiences

The Adverse Childhood Experience (ACE) scale, initially created through the Kaiser-CDC study [20], was employed to assess ACEs. This instrument consists of ten items encompassing three categories of experiences before age 18: household dysfunction, neglect, and abuse. Each “yes” response was scored as one point, with the sum of affirmative answers representing the total ACE score. For analysis, the total score was dichotomized, with a score of 0 coded as “0” and any score coded as “1”. The scale has demonstrated satisfactory reliability and validity in Chinese populations [33, 34], and in this study, the Cronbach’s alpha was 0.695.

### Self-efficacy

The General Self-efficacy Scale (GSES), created by Zhang and Schwarzer [35], is a widely recognized tool to assess self-confidence [36]. The scale comprises 10 items, each rated on a 4-point scale, resulting in total scores between 10 and 40. The Chinese adaptation has been shown to possess strong consistency and accuracy [37, 38] and has been effectively employed in our previous research [31, 39, 40]. In the current study, the Cronbach’s alpha values ranged from 0.940 to 0.943.

### Depression

The Hospital Anxiety and Depression Scale (HADS), initially created by Zigmond and Snaith [41], consists of 14 items designed to assess levels of anxiety and depression. For this study, only the depression subscale (comprising 7 items) was utilized. Each item is rated on a 4-point scale (0–3), resulting in a total possible score between 0 and 21, with higher scores reflecting more severe depressive symptoms [42]. The Chinese adaptation of HADS-D has demonstrated good reliability [43], and in this research, Cronbach’s alpha values ranged from 0.698 to 0.716.

### Statistical analyses

Before formal analysis, participants lost to follow-up or with missing data were excluded to ensure the integrity and validity of the data. Descriptive analyses were first used to characterize the covariates and ACEs. Binary logistic regression was then employed to investigate the differences in general information based on the presence or absence of ACEs. Correlation analysis was conducted to examine the relationships between self-efficacy (T1-T3) and prenatal depression (T1-T3).

Subsequently, the time-varying impact of self-efficacy on prenatal depression was estimated by a single parallel process LGCM. The model fit was assessed using the *χ2* test, root mean square error of approximation (RMSEA), Tucker-Lewis Index (TLI), and comparative fit index (CFI) [44]. A good model fit was indicated by CFI and TLI values greater than 0.95, and an RMSEA value below 0.08 was considered acceptable [44].

Lastly, to further investigate whether the longitudinal link between self-efficacy and prenatal depression varied according to ACEs exposure, multigroup LGCM analyses were conducted [45].

All statistical analyses were conducted using SPSS (version 26.0), Mplus (version 8.1), and JASP (version 0.18.3.0). Statistical significance was established with a *P* value less than 0.05, and standardized regression coefficients (*β*) were reported for model results.

### Ethics

All procedures adhered to the Declaration of Helsinki. Ethics approval was granted by the institutional review board of the participating hospital (Number: K-2022-024). This research formed part of the Be Resilient to Postpartum Depression (BRPD) initiative (Registration number: ChiCTR2100048465, registered on 09/07/2021). Written informed consent was secured from every participant before the investigation. To ensure participant privacy and confidentiality, all data were anonymized before analysis, and no personally identifiable information was collected or stored.

## Results

### Descriptive analyses

**Fig. 1:**
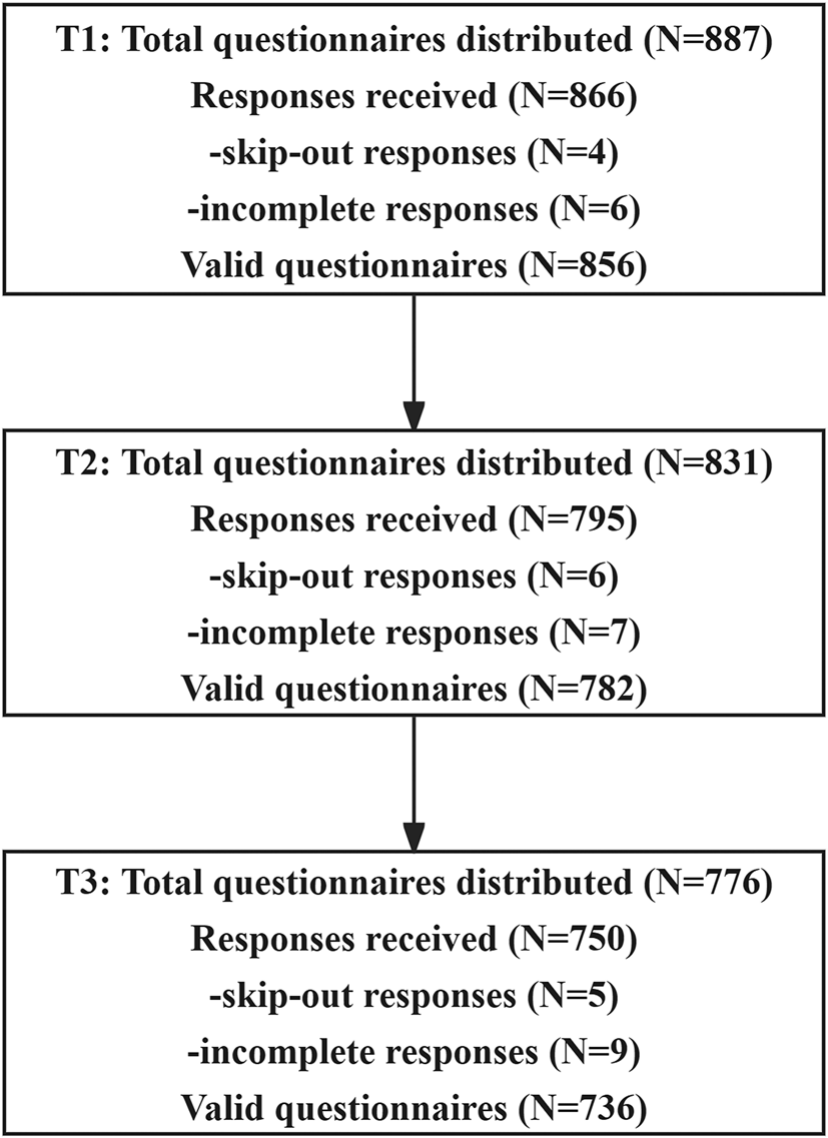
The flowchart of data collection

**Fig. 2:**
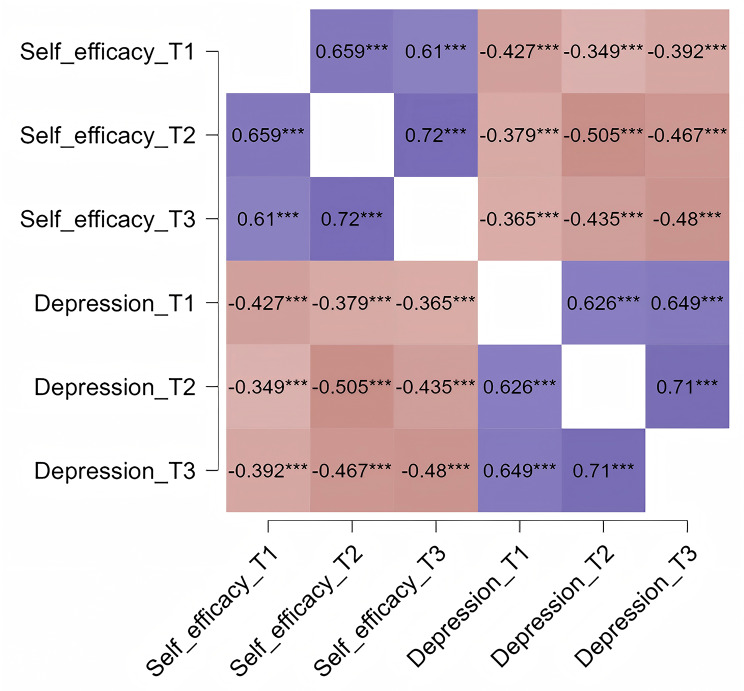
Correlations between variables, Note: *** *P* < 0.001

As illustrated in Fig. 1, a total of 887 questionnaires were distributed at T1, resulting in 866 responses. However, 4 of these were categorized as part of the skip-out pattern, and 6 were not fully completed, yielding 856 valid responses and a validity rate of 96.5%. In T2, 831 questionnaires were distributed, with 795 responses received. Among these, 6 were identified as skip-out responses, and 7 were incomplete, leading to 782 valid responses and a validity rate of 94.1%. For T3, 776 questionnaires were distributed, and 750 responses were returned. Of these, 5 fell under the skip-out category, and 9 were not fully completed, resulting in 736 valid responses and a validity rate of 94.7%. Across all three rounds, a total of 721 valid questionnaires were matched. Demographic characteristics collected during T1 indicated a mean age of 29.72 years (SD = 4.02). Over 90% of participants were married. About 70% participants came from households with a monthly average income above 4,000 RMB or were in employment, while over half were nulliparous. Additional details are provided in Table 1. No significant differences in basic information were observed between participants with and without ACEs (Table S1). Correlation analysis showed significant associations between self-efficacy and depression at each measurement occasion (*P* < 0.001; see Fig. 2).

**Table 1:** Baseline and follow-up characteristics

Characteristics	Participants
Baseline (T1)	
Age, M(SD)	29.72 (4.04)
Education level, n (%)	
High school or less	234 (32.5)
Junior college degree	252 (35.0)
Bachelor or above	235 (32.5)
Marital status, n (%)	
Married	677 (93.9)
Unmarried	44 (6.1)
Work status, n (%)	
Employed	525 (67.9)
Unemployed	196 (32.1)
Monthly average household income, n (%)	
≤ 4000 RMB	181 (25.1)
>4000 RMB	540 (74.9)
Parity, n(%)	
Multiparous	343 (47.6)
Nulliparous	378 (52.4)
Adverse childhood experiences, n (%)	
Yes	134 (18.6)
No	587 (81.4)
Self-efficacy, M(SD)	26.18 (6.63)
Depression, M(SD)	3.94 (2.78)
Follow-up (T2)	
Self-efficacy, M(SD)	25.77 (6.19)
Depression, M(SD)	4.23 (2.73)
Follow-up (T3)	
Self-efficacy, M(SD)	25.50 (5.98)
Depression, M(SD)	4.68 (2.77)

### Joint trajectory analysis of self-efficacy and prenatal depression

**Fig. 3:**
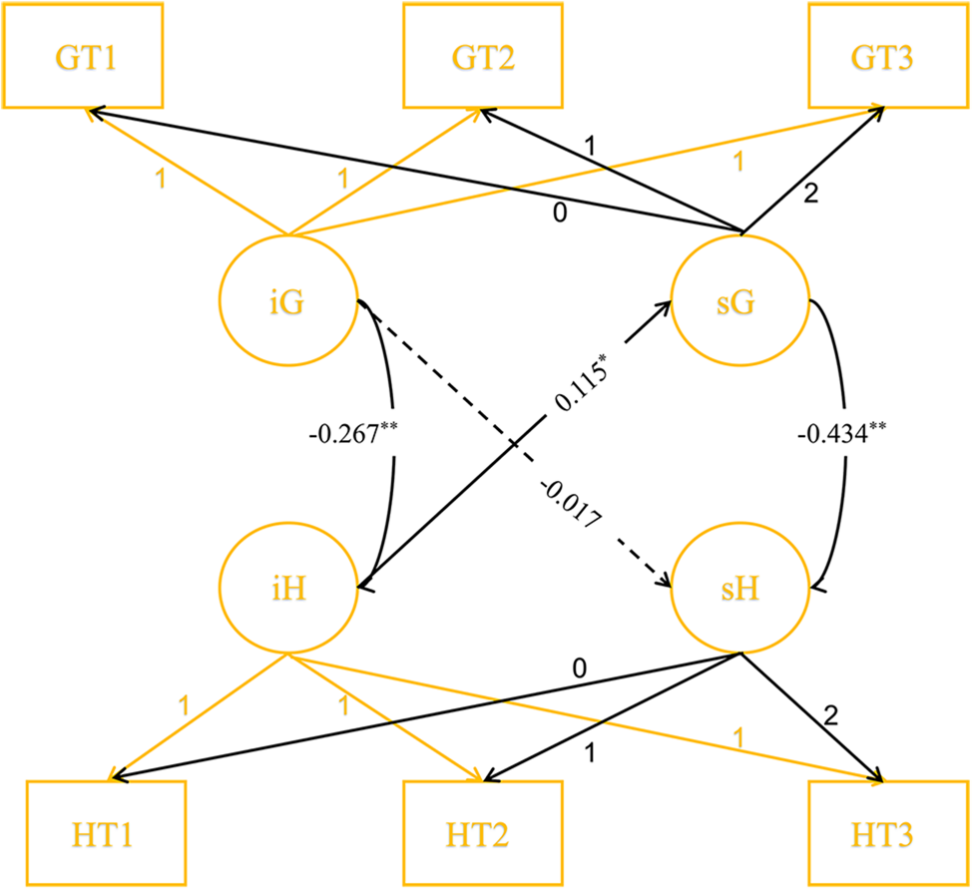
PP-LGCM for GSES and HADS-D (whole sample). Note: *P<0.05,**P<0.01, G: GSES, H: HADS-D, i -Intercept, s-Slope

The parallel process LGCM demonstrated satisfactory fit to the data (*χ*2 = 93.802, *df* = 24, TLI = 0.943, CFI = 0.970, RMSEA = 0.064). As illustrated in Fig. 3, baseline GSES scores exhibited a negative association with baseline HADS-D scores (*β*=-0.267, *P* < 0.01). Additionally, the rate of change in GSES scores was negatively associated with the rate of change in HADS-D scores (*β*=-0.434, *P* < 0.01). However, baseline GSES scores did not significantly predict the change rate of HADS-D scores (*β*=-0.017, *P* = 0.097). Conversely, baseline HADS-D scores significantly predicted the change rate of GSES scores (*β* = 0.115, *P* = 0.034).

### Multi-group joint trajectory analysis of self-efficacy and prenatal depression

**Fig. 4:**
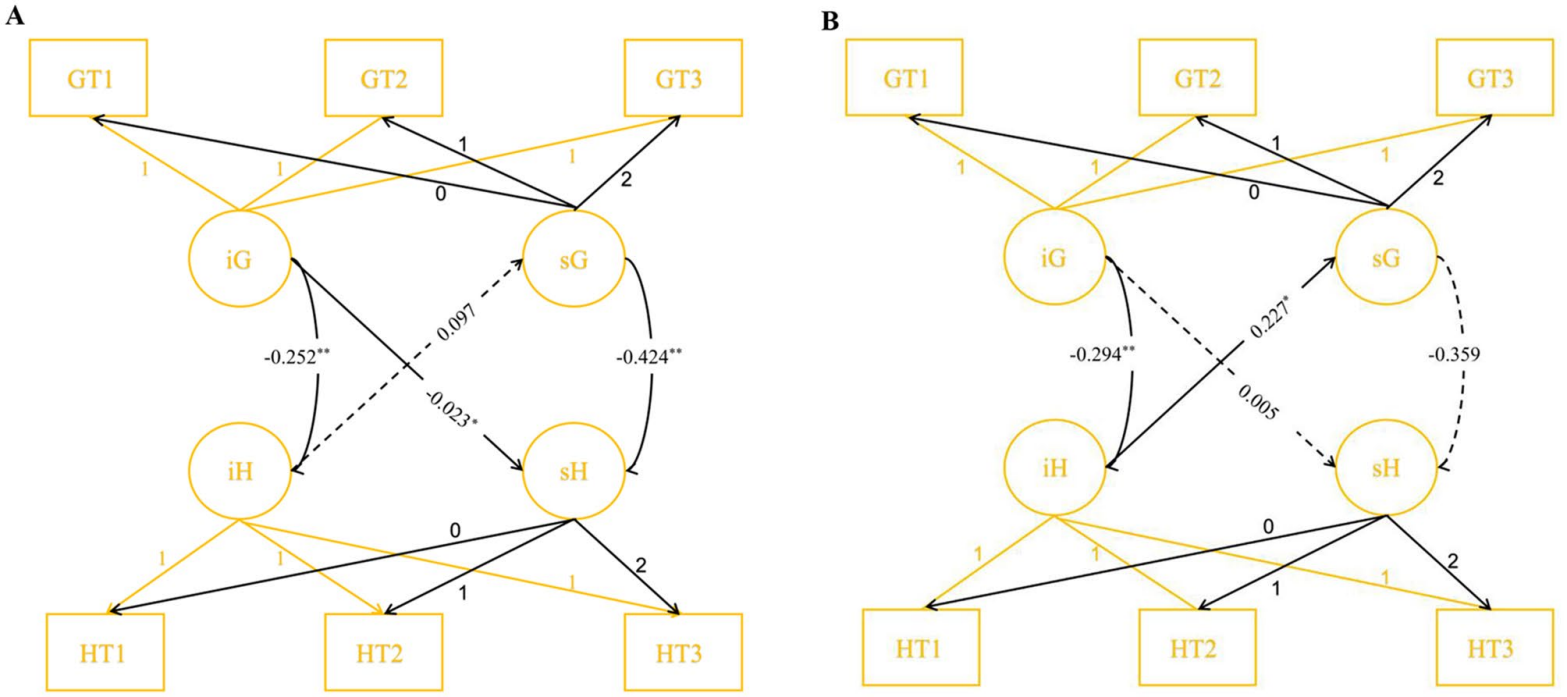
(**A**) PP-LGCM for GSES and HADS-D (without ACEs). (**B**) PP-LGCM for GSES and HADS-D (with ACEs). Note: *P<0.05, **P<0.01, G: GSES, H: HADS-D, i -Intercept, s-Slope

To explore whether the longitudinal trajectories of self-efficacy and prenatal depression differed between pregnant women with and without ACEs, we introduced ACEs as a covariate. The model demonstrated good model fit (*χ*2 = 112.194, *df* = 48, TLI = 0.948, CFI = 0.972, RMSEA = 0.061). In the group without ACEs (Fig. 4-A), baseline GSES scores negatively correlated with baseline HADS-D scores (*β*=-0.252, *P* < 0.01). The change rate of GSES scores negatively correlated with the rate of changes in HADS-D scores (*β*=-0.424, *P* < 0.01). Additionally, baseline GSES scores significantly predicted the change rate of HADS-D scores (*β*=-0.023, *P* = 0.031), while baseline HADS-D scores did not significantly predict the change rate of GSES scores (*β* = 0.097, *P* = 0.131). Conversely, in the group with ACEs (Fig. 4-B), baseline GSES scores were negatively associated with baseline HADS-D scores (*β*=-0.294, *P* < 0.01). However, the rate of change in GSES scores was not significantly associated with the rate of change in HADS-D scores (*β*=-0.39, *P* = 0.214). Baseline GSES scores did not significantly predict the change rate of HADS-D scores (*β* = 0.005, *P* = 0.880). In contrast, baseline HADS-D scores significantly predicted the change rate of GSES scores (*β* = 0.227, *P* = 0.049).

## Discussion

The present study offers valuable insights into the complex link between self-efficacy and prenatal depression from the first to the third trimester, while also exploring the moderating role of ACEs. Though latent growth curve models have been used to investigate prenatal depression in pregnant women in China and other countries [46, 47], they have primarily focused on the trajectories of depressive symptoms without considering the influence of other variables’ trajectories. By incorporating the trajectories of self-efficacy alongside those of depression, our study reveals a more dynamic relationship that highlights the reciprocal influences between these constructs.

The observed inverse connection between initial self-efficacy and baseline depression highlights the significance of self-efficacy as a psychological resource for pregnant women. Those with higher self-efficacy tend to perceive challenges as manageable and adopt proactive problem-solving strategies, thereby lowering their susceptibility to depressive symptoms [48, 49]. This aligns with Bandura’s theory of self-efficacy, which suggests that belief in one’s capabilities enhances resilience and coping strategies, serving as a protective factor against depression [50].

Furthermore, the significant association between the change rates of self-efficacy and depression underscores a dynamic relationship where fluctuations in self-efficacy directly influence emotional well-being. This highlights the importance of maintaining self-efficacy throughout pregnancy. According to Social Cognitive Theory, self-efficacy is shaped by personal experiences and social influences [51]. Positive experiences, such as effectively managing or receiving supportive feedback from healthcare providers, can bolster self-efficacy and enhance emotional well-being [52, 53]. Conversely, negative experiences, including complications or insufficient support, can undermine self-efficacy, heightening the risk of prenatal depression [49, 54]. Programs aimed at continuously enhancing self-efficacy during pregnancy may help women sustain a positive mental health trajectory. Techniques such as skills training [55], supportive counseling [56], and mindfulness practices [57] have shown promise in fostering self-efficacy and alleviating depressive symptoms.

Additionally, baseline depression levels significantly affect the rate of change in self-efficacy, indicating an important interaction between these constructs. The Diathesis-Stress Model suggests that depressive symptoms, once triggered, can diminish self-efficacy through the interplay of vulnerability and stress, leading to feelings of helplessness and a reluctance to engage in health-promoting behaviors [58]. Prior research has documented cognitive control deficits and motivational impairments in individuals with depression, which are essential for maintaining self-efficacy [59]. These deficits hinder the ability to set and pursue goals effectively, thus impairing adaptive coping mechanisms [60, 61]. Thus, implementing early screening for depression during the first trimester is crucial for enabling timely interventions and support [62].

The multi-group analysis reveals that the link between self-efficacy and prenatal depression is significantly affected by the presence of ACEs. The emotional aftermath of ACEs can evoke feelings of inadequacy and anxiety, which may overshadow the protective effects of high self-efficacy [63]. This concern is particularly relevant during pregnancy, as hormonal fluctuations can amplify emotional responses and exacerbate feelings of helplessness [64]. Furthermore, psychological scars from past traumas can undermine emotional resilience, increasing vulnerability to stressors during this critical period [65, 66]. This vulnerability has biological underpinnings, as chronic stress related to ACEs can alter the functioning of the hypothalamic-pituitary-adrenal axis, resulting in increased cortisol production that adversely affects cognitive function and mood [67]. In cultures such as China, where maintaining family honor and emotional restraint are emphasized, individuals may be more likely to internalize stress associated with ACEs, further exacerbating this biological disruption [68]. Given these complexities, pregnant women with ACEs would benefit from tailored support that directly addresses their trauma history while also fostering self-efficacy.

### Limitations

There are several limitations to this study that warrant consideration. Firstly, the sample was recruited from a single region, which may restrict the generalizability of the findings to other populations or cultural settings. Secondly, the use of self-reported questionnaires may lead to response bias, as participants might either minimize or exaggerate their symptoms due to social desirability or limited self-awareness [69]. Additionally, the ACE subgroup comprised only approximately 130 participants, hence suggesting that the observed differences in coefficients may be influenced by this insufficient sample size. Finally, while we examined ACEs, other contextual factors (i.e., pre-existing conditions, pregnancy-related complications, changes in body shape due to weight gain), as well as additional demographic variables, were not assessed, which may influence self-efficacy and prenatal depression.

### Implications

The study’s findings offer valuable insights for clinical practice and intervention development. Programs aimed at enhancing self-efficacy among pregnant women could be instrumental in reducing prenatal depression. Evidence from randomized controlled trials indicates that antenatal education focused on health literacy [70], as well as health education programs utilizing motivational interviewing [71], positively improve general self-efficacy among pregnant women. Addressing ACEs is equally crucial. Integrating trauma-informed care into prenatal services can enhance the overall well-being of pregnant women, particularly those with a history of adverse experiences. Antenatal care settings can utilize the standard ACE questionnaire, such as the one employed in this study [20], or implement customized ACE screening tools tailored specifically for the context of pregnancy [72]. For pregnant women identified with significant trauma histories or symptoms, targeted interventions should be introduced to mitigate the lasting effects of ACEs and enhance perinatal outcomes. For example, women can be taught to recognize trauma patterns and apply guided imagery techniques to connect with their inner strengths, thereby fostering a greater sense of control and confidence [73].

## Conclusions

In conclusion, our findings elucidate the complex relationships among self-efficacy, prenatal depression, and adverse childhood experiences in pregnant women. These results underscore the necessity for tailored interventions that simultaneously address both self-efficacy and past traumas, which are essential for effectively supporting women during this transformative and often challenging period of their lives.

## Supplementary Information

Below is the link to the electronic supplementary material.

Supplementary Material 1

## Data Availability

The data that support the findings of this study are available from the corresponding author upon reasonable request.
