# Supplementary material for: The relationship between self-efficacy and prenatal depression in Chinese pregnant women: a parallel latent growth curve model
Source: BMC Psychiatry. 2025 Nov 28;25:1202. doi: 10.1186/s12888-025-07623-4 (PMC12752194; doi:10.1186/s12888-025-07623-4)
Supplement: Supplementary file 1 — Supplementary Material 1 [file 12888_2025_7623_MOESM1_ESM.docx]

| **Table S1 Comparison of general information between groups with and without ACEs** | | |
| --- | --- | --- |
| Characteristics | Participants | *P* value |
| Age, M(SD) | 29.72(4.04) | 0.113 |
| Academic degree, n (%) |  |  |
| High school or less | 234 (32.5) | 0.245 |
| Junior college degree | 252 (35.0) |  |
| Bachelor or above | 235 (32.5) |  |
| Marital status, n (%) |  |  |
| Married | 677 (93.9) | 0.943 |
| Unmarried | 44 (6.1) |  |
| Employment, n (%) |  |  |
| Yes | 525 (67.9) | 0.902 |
| No | 196 (32.1) |  |
| Monthly average household income, n (%) |  |  |
| ≤4000 RMB | 181 (25.1) | 0.888 |
| ＞4000 RMB | 540 (74.9) |  |
| Parity, n (%) |  |  |
| Multiparous | 343 (47.6) | 0.598 |
| Nulliparous | 378 (52.4) |  |
